# Risk Factors and Outcomes of Hospital Acquired Pneumonia in Young Bangladeshi Children

**DOI:** 10.3390/life11101030

**Published:** 2021-09-30

**Authors:** Abu Sadat Mohammad Sayeem Bin Shahid, Tahmina Alam, Lubaba Shahrin, K. M. Shahunja, Md. Tanveer Faruk, Mst. Mahmuda Ackhter, Ishrat Jahan Karim, Shafiul Islam, Mostafa Taufiq Ahmed, Haimanti Saha, Irin Parvin, Tahmeed Ahmed, Mohammod Jobayer Chisti

**Affiliations:** 1 International Centre for Diarrhoeal Disease Research, Bangladesh, Dhaka 1212, Bangladesh; drtahmina@icddrb.org (T.A.); lubabashahrin@icddrb.org (L.S.); tanveer.faruk@icddrb.org (M.T.F.); mahmuda.ackhter@icddrb.org (M.M.A.); haimanti@icddrb.org (H.S.); irin.parvin@icddrb.org (I.P.); tahmeed@icddrb.org (T.A.); chisti@icddrb.org (M.J.C.); 2 The University of Queensland, Brisbane, QLD 4072, Australia; k.shahunja@uq.net.au; 3 Sylhet MAG Osmani Medical College Hospital, Sylhet 3100, Bangladesh; drijkog@gmail.com (I.J.K.); drtaufiqns@gmail.com (M.T.A.); 4 World Health Organization, Sylhet 3100, Bangladesh; shafiulpiyal@gmail.com

**Keywords:** risk factors, outcome, hospital acquired pneumonia, Bangladesh, children, persistent diarrhea, severe acute malnutrition, bacteremia, hypoxemia

## Abstract

Hospital acquired pneumonia (HAP) is common and often associated with high mortality in children aged five or less. We sought to evaluate the risk factors and outcome of HAP in such children. We compared demographic, clinical, and laboratory characteristics in children <5 years using a case control design during the period of August 2013 and December 2017, where children with HAP were constituted as cases (*n =* 281) and twice as many randomly selected children without HAP were constituted as controls (*n =* 562). HAP was defined as a child developing a new episode of pneumonia both clinically and radiologically after at least 48 h of hospitalization. A total of 4101 children were treated during the study period. The mortality was significantly higher among the cases than the controls (8% vs. 4%, *p =* 0.014). In multivariate logistic regression analysis, after adjusting for potential confounders, it was found that persistent diarrhea (95% CI = 1.32–5.79; *p =* 0.007), severe acute malnutrition (95% CI = 1.46–3.27; *p* < 0.001), bacteremia (95% CI = 1.16–3.49; *p =* 0.013), and prolonged hospitalization of >5 days (95% CI = 3.01–8.02; *p* < 0.001) were identified as independent risk factors for HAP. Early identification of these risk factors and their prompt management may help to reduce HAP-related fatal consequences, especially in resource limited settings.

## 1. Introduction

Hospital acquired pneumonia (HAP) is common and often associated with high mortality [1]. To meet the World Health Organization’s (WHO) Integrated Global Action Plan for the Prevention and Control of Pneumonia and Diarrhoea (GAPPD), i.e., to reduce deaths from pneumonia to less than 3 children per 1000 live births by 2025, we need to continue an 8% average annual rate of reduction in pneumonia-related deaths in Bangladesh [2]. Thus, we need to reduce HAP related mortality in order to achieve GAPPD. In fact, HAP often occurs as a ramification of longer hospitalization, causes an overuse of health care resources—such as hospital beds, food, care of doctors and nurses—and HAP often becomes responsible for increased hospitalization costs [1]. In order to reduce the incidence of HAP as well as its hospital-related costs, we need to understand the risk factors of HAP. Thus, we aimed to evaluate the risk factors and outcomes of HAP in children aged five or less.

## 2. Materials and Methods

### 2.1. Study Site

The study was conducted in the Dhaka Hospital of the International Centre for Diarrhoeal Disease Research, Bangladesh. The hospital is a tertiary facility that serves the greater Dhaka metropolitan area and provides specialized care for diarrheal illness as well as childhood respiratory illness and undernutrition [3].

### 2.2. Study Population and Design

Using discharge certificates from the electronic database of the hospital, all children under 5 years of age between August 2013 and December 2017 were evaluated for HAP. We compared demographic, clinical, and laboratory characteristics on admission between the children having HAP (cases) and without HAP (controls). For an unmatched case-control analysis, twice as many children without HAP were randomly selected as the controls. For randomization we used SPSS for Windows (version 20.0). All the study children were managed following standard treatment according to hospital guidelines that have been described in detail previously [3]. HAP was defined if a child developed a new episode of pneumonia both clinically and radiologically at least 48 h after hospitalization [1].

### 2.3. Measurements

A case report form was designed for this study to extract demographic (age and male gender), clinical (persistent diarrhea; fever, dehydration; severe acute malnutrition; hypoxemia; congenital heart disease, including if a child had a murmur on auscultation on admission or had echocardiographically confirmed heart disease; unconsciousness; severe sepsis; anemia) and laboratory data (bacteremia, white blood cell count/cu mm, hemoglobin %) (Table 1). We used standard definitions of variables in Table 1 that include persistent diarrhea [4], dehydration [4], severe acute malnutrition [4], hypoxemia [4], severe sepsis [3], bacteremia [3], and anemia [4].

### 2.4. Blood Cultures and Antimicrobial Sensitivity Testing

Two mL of fresh venous blood were collected from the patients with all aseptic precautions and were seeded directly into BacT/ALERT culture bottles. They were loaded into the BacTAlert 3D system. Only one blood sample for each of the participants was collected and tested because of resource constraints.

Antibiotic sensitivity testing was carried out using disk diffusion as recommended by the Clinical Laboratory Standards Institute (CLSI). During the study period, the laboratory followed the available updated editions of the CLSI guidelines (CLSI-2014, CLSI-2015, CLSI-2016, CLSI-2017) [5]. Commercial antimicrobial discs (Oxoid, Basingstoke, United Kingdom) were used for the antibiotic sensitivity test. The zone of inhibition was measured by using the CLSI guidelines. Minimum inhibitory concentrations (MICs) were not performed due to limited resources. All reports of culture and sensitivity were available by 48–72 h of sample collection.

### 2.5. Statistical Analysis

Data were analyzed with SPSS for Windows, version 20.0 (IBM Corp, New York, NY, USA) and STATA, version 13. Differences in proportions were compared using the chi-square test or Fisher’s exact test, as appropriate, and differences in the means were compared by the Student’s *t*-test. The Mann–Whitney *U* test compared data that were not normally distributed. A probability of less than 0.05 at a 95% confidence interval (CI) was considered statistically significant. The strength of associations was determined by calculating the odds ratios and their 95% CIs.

Those who had significant associations with HAP were put into the multivariate logistic regression model, where HAP was the dependent variable and the factors significantly associated with HAP were the independent variables.

## 3. Results

A total of 4101 children received treatment during the study period. Among them 281 (7%) had HAP and the rest did not have HAP, from which latter group we took 562 randomly selected children who served as the comparison group. In bivariate analysis we had found that children aged five or less with HAP more often presented with persistent diarrhea (OR: 5.31, 95% CI = 3.01–9.36), severe acute malnutrition (OR: 2.35, 95% CI = 1.75–3.14), hypoxemia (OR: 1.44, 95% CI = 1.06–1.96), bacteremia (OR: 1.78, 95% CI = 1.08–2.92), and had prolonged hospitalization of >5 days (OR: 6.24, 95% CI = 4.33–9.00), compared to those without HAP. The mortality rate was also significantly higher among the children with HAP compared to those without HAP (8% vs. 4%; OR: 2.09, 95% CI = 1.16–3.77; *p =* 0.014) (Table 1). 

In multivariate logistic regression analysis, after adjusting for the potential confounders, it was shown that persistent diarrhea (OR: 2.77, 95% CI = 1.32–5.79), severe acute malnutrition (OR: 2.18, 95% CI = 1.46–3.27), bacteremia (OR: 2.01, 95% CI = 1.16–3.49), and prolonged hospitalization of >5 days (OR: 4.91, 95% CI = 3.01–8.02) were the independent risk factors for HAP in children aged five or less (Table 2).

The most common bacterial pathogens identified among the children with HAP were coagulase-negative *staphylococcus* (CNS) followed by *Staphylococcus haemolyticus*, *Micrococcus* species, *Acinetobacter*, *Pseudomonas* and *Escherichia* coli and among the comparison groups these were *Pseudomonas* followed by *Micrococcus* species, CNS, *Escherichia* coli and *Staphylococcus haemolyticus* (Table 3). However, the distribution of individual bacterial pathogen between the groups was comparable. The antibiotic-resistant pattern between the groups is shown in Table 3.

## 4. Discussion

This is the very first study that specifically observed the independent association of persistent diarrhea with HAP. According to the WHO, if in a child diarrhea starts acutely with or without blood and continues for 14 days or more, it is termed as persistent diarrhea [4]. A recent study involving children with persistent diarrhea revealed that in most of the cases children require prolonged hospitalization (>5 days) and become malnourished [6]. In our study, we found an association with HAP among children with severe acute malnutrition and prolonged hospitalization (>5 days). Thus, our observation of association of persistent diarrhea with HAP is quite understandable.

The observation of association of severe acute malnutrition with HAP is also explicable. Severely malnourished children are often immune compromised and vulnerable to infectious pathogens, especially respiratory bacterial pathogens causing pneumonia. The findings are consistent with earlier study observations [7]. The observation of prolonged hospitalization (>5 days) is also consistent with a number of earlier studies [1,8]. Bacteremia predominated by CNS and Gram negatives was found to be independently associated with HAP, which is not surprising and a number of previous studies had the same observations [1,8]. Although bacteremia was more often observed among the children with HAP, the spectrum and frequency of bacterial pathogens between the children with and without HAP were comparable. A number of previous studies showed the predominance of CNS and Gram negative bacterial pathogens in children with pneumonia and almost all of them also had severe malnutrition [9,10,11]. For Gram negatives, we may speculate this observation might be due to breaches of normal flora of the intestinal wall in diarrheal children that harbor Gram negatives, such as *Pseudomonas, Escherichia* coli, and *Klebsiella,* and potential insults to the normal flora during diarrhea that may allow translocation of these bacteria from the normal flora of the gut to systemic infection. Moreover, the role of potential translocation of Gram negatives in children with severe acute malnutrition having strong association with HAP could not be ruled out.

The observation of a high resistance pattern of bacterial isolates from blood predominated by CNS and Gram negatives, both in children with and without HAP in our study children, to WHO-recommended first line (amoxicillin/ampicillin and gentamicin) and second line (ceftriaxone) antibiotics for the treatment of severe pneumonia [4] is alarming. The policymakers may need to focus in this issue and revise the guidelines for the management of these children.

As children having HAP in our study were strongly associated with severe acute malnutrition and bacteremia and as both the entities were found to have strong association with mortality [12,13], the observation of higher deaths among children with HAP compared to those without HAP is understandable and consistent with the findings of a number of earlier studies [7,14].

The main limitation of the study is its retrospective nature, which might have limited our ability to further evaluate potential variables of interest that would have an association with HAP. The strength of this study is the reporting of children with HAP having the co-morbidity of diarrhea, the leading two causes of global mortality in children under five years of age.

## 5. Conclusions

The results of our study revealed that HAP is common in children less than five years of age and associated with higher case-fatality rates compared to those without HAP. Children less than five years having persistent diarrhea, severe acute malnutrition, bacteremia, and requiring prolonged hospitalization >5 days were prone to develop HAP. Early identification of these risk factors and their prompt management may help to reduce HAP-related fatal consequences, especially in resource-limited settings. However, prospective research with a large sample is imperative to accept or refute our observations.

## Figures and Tables

**Table 1:** Characteristics of children under 5 years of age with hospital acquired pneumonia compared to those without hospital acquired pneumonia.

Characteristics	Children with Hospital Acquired Pneumonia (*n =* 281) (%)	Children without Hospital Acquired Pneumonia (*n =* 562) (%)	OR	95% CI	*p*-Value
***Predicting factors of hospital acquired pneumonia***					
Male gender	194 (69)	364 (65)	1.21	0.89–1.65	0.217
Age in months (median, IQR)	8.0 (5.43, 10.08)	8.43 (4.68, 11.13)	-	-	0.678
Immunization as per EPI schedule	189 (67)	344 (61)	1.22	0.79–1.89	0.363
Persistent diarrhea	42 (15)	18 (3)	5.31	3.01–9.36	<0.001
Fever	157 (56)	315 (56)	0.99	0.74–1.32	0.961
Dehydration (some/severe)	70 (25)	120 (21)	1.22	0.87–1.71	0.244
Severe acute malnutrition	165 (59)	212 (38)	2.35	1.75–3.14	<0.001
Hypoxemia	96 (34)	149 (27)	1.44	1.06–1.96	0.021
Congenital heart disease	21 (8)	37 (7)	1.15	0.66–1.99	0.631
Unconsciousness	13 (5)	37 (7)	0.67	0.35–1.27	0.221
Severe sepsis	24 (9)	46 (8)	1.05	0.63–1.75	0.860
Bacteremia	44/227 (19)	29/234 (12)	1.78	1.08–2.92	0.023
White blood cell count/cu mm(median, IQR)	14,205 (10,327, 18,195)	14,450 (10,048, 17,850)	-	-	0.459
Hemoglobin (%) (mean, SD)	10.56 (2.21)	10.55 (1.99)	0.10	−0.30–0.32	0.947
Anemia (Hb% < 9.3 gm/dl)	71/265 (27)	108/476 (23)	1.25	0.88–1.76	0.211
Prolonged hospitalization (hospital stay >5 days)	239 (85)	268 (48)	6.24	4.33–9.00	<0.001
***Outcomes during hospitalization after development of hospital acquired pneumonia***					
Total duration of hospital stay (median, IQR)	11.0 (7.0, 15.0)	5.0 (3.0, 7.0)	-	-	<0.001
Deaths	23 (8)	23 (4)	2.09	1.16–3.77	0.014

OR: odds ratio; CI: confidence interval; IQR: interquartile range; SD: standard deviation; Hb: hemoglobin; EPI: extended program on immunization.

**Table 2:** Results of logistic regression analysis to explore the independent predicting factors for hospital acquired pneumonia.

Characteristics	OR	95% CI	*p*-Value
Persistent diarrhea	2.77	1.32–5.79	0.007
Severe acute malnutrition	2.18	1.46–3.27	<0.001
Hypoxemia	0.98	0.63–1.51	0.915
Bacteremia	2.01	1.16–3.49	0.013
Prolonged hospitalization (Hospital stay >5 days)	4.91	3.01–8.02	<0.001

OR: odds ratio; CI: confidence interval.

**Table 3:** Bacterial isolates from blood and their antibiotic-resistant pattern in children less than five years of age with and without hospital acquired pneumonia (HAP).

Isolates	Total No. of Isolates: HAP (*n =* 40) vs. without HAP (*n =* 29) (%)	Resistance to Antibiotics (HAP vs. without HAP)								
		AMX	GEN	CRO	AZM	CIP	CFM	CAZ	AMK	LVX
Coagulase-negative staphylococcus (CNS)	15 (37) vs. 7 (24)	9/12 (75) vs. 2/4 (50)	4/14 (29) vs. 0/4 (0)	8/9 (89) vs. 0/4 (0)	-	9/14 (64) vs. 1/4 (25)	9/14 (64) vs. 0/0 (0)	1/1 (100) vs. 0/0 (0)	0/1 (0) vs. 0/0 (0)	-
Acinetobacter species	3 (7) vs. 1 (3)	3/3 (100) vs. 1/1 (100)	1/3 (33) vs. 0/1 (0)	3/3 (100) vs. 1/1 (100)	2/2 (100) vs. 1/1 (100)	3/3 (100) vs. 1/1 (100)	3/3 (100) vs. 1/1 (100)	2/3 (67) vs. 1/1 (100)	1/3 (33) vs. 0/1 (0)	-
Pseudomonas species	2 (5) vs. 7 (24)	0/0 (0) vs. 0/0 (0)	0/2 (0) vs. 1/5 (20)	1/1 (100) vs. 1/5 (20)	0/0 (0) vs. 0/0 (0)	0/2(0) vs. 0/6 (0)	0/2 (0) vs. 0/0 (0)	0/2 (0) vs. 1/6 (17)	0/2 (0) vs. 2/6 (33)	0/1 (0) vs. 0/1 (0)
Klebsiella	1 (2) vs. 1 (3)	1/1 (100) vs. 1/1 (100)	1/1 (100) vs. 1/1 (100)	1/1 (100) vs. 1/1 (100)	1/1 (100) vs. 1/1 (100)	1/1 (100) vs. 1/1 (100)	1/1 (100) vs. 1/1 (100)	0/1 (0) vs. 1/1 (100)	0/1(0) vs. 1/1 (100)	-
Pneumococcus	1 (2) vs. 0 (0)	-	-	0/1 (0) vs. 0/0 (0)	1/1 (100) vs. 0/0 (0)	-	-	0/1 (0) vs. 0/0 (0)	0/1 (0) vs. 0/0 (0)	0/1 (0) vs. 0/0 (0)
Salmonella typhi	1 (2) vs. 1 (3)	0/1 (0) vs. 0/1 (0)	0/1 (0) vs. 0/0 (0)	0/1 (0) vs. 0/1 (0)	0/1 (0) vs. 0/1 (0)	1/1 (100) vs. 1/1 (100)	1/1 (100) vs. 0/1 (0)	0/1 (0) vs. 0/0 (0)	0/1 (0) vs. 0/0 (0)	-
Staphylococcus haemolyticus	6 (15) vs. 4 (14)	5/5 (100) vs. 3/3 (100)	5/6 (83) vs. 3/4 (75)	5/5 (100) vs. 3/4 (75)	-	6/6 (100) vs. 2/4 (50)	6/6 (100) vs. 0/0 (0)	-	-	-
Escherichia coli	2 (5) vs. 4 (14)	2/2 (100) vs. 3/4 (75)	1/2 (50) vs. 0/4 (0)	2/2 (100) vs. 2/4 (50)	1/1 (100) vs. 4/4 (100)	2/2 (100) vs. 3/4 (75)	2/2 (100) vs. 2/3 (67)	2/2 (100) vs. 3/4 (75)	1/1 (100) vs. 0/4 (0)	-
Enterococcus spp.	2 (5) vs. 0 (0)	1/2 (50) vs. 0/0 (0)	1/2 (50) vs. 0/0 (0)	1/1 (100) vs. 0/0 (0)	-	2/2 (100) vs. 0/0 (0)	2/2 (100) vs. 0/0 (0)	-	-	-
Corynebacterium spp.	1 (2) vs. 0 (0)	-	0/1 (0) vs. 0/0 (0)	0/1 (0) vs. 0/0 (0)	-	1/1 (100) vs. 0/0 (0)	1/1 (100) vs. 0/0 (0)	-	-	-
Micrococcus species	6 (15) vs. 5 (17)	0/0 (0) vs. 0/1 (0)	0/1 (0) vs. 0/1 (0)	0/1 (0) vs. 0/1 (0)	-	0/1 (0) vs. 0/1 (0)	0/1 (0) vs. 0 (0)	-	-	-
Non-typhoidal salmonella	0 (0) vs. 1 (3)	0/0 (0) vs. 0/1 (0)	-	0/0 (0) vs. 0/1 (0)	0/0 (0) vs. 1/1 (100)	0/0 (0) vs. 1/1 (100)	0/0 (0) vs. 0/1 (0)	-	-	-
Streptococcus species	0 (0) vs. 1 (3)	-	-	0/0 (0) vs. 0/1 (0)	0/0 (0) vs. 1/1 (100)	-	-	-	-	0/0 (0) vs. 1/1 (100)

AMX, amoxycillin; GEN, gentamicin; CRO, ceftriaxone; AZM, azythromycin; CIP, ciprofloxacin; CFM, cefotaxime; CAZ, ceftazidime; AMK, amikacin; LVX, levofloxacin.

## Data Availability

The dataset contained personal information of the study participants. Our institutional review board will not disclose this kind of information. Thus, our policy is not to make available the dataset in the manuscript, the supplemental files, or a public repository. However, data related to this manuscript are available upon request and researchers who meet the criteria for access to confidential data may contact with Ms. Armana Ahmed (aahmed@icddrb.org) to the research administration of icddrb [http://www.icddrb.org/ (accessed on 25 August 2021)].
